# Enhanced cycle stability of a NiCo_2_S_4_ nanostructured electrode for supercapacitors fabricated by the alternate-dip-coating method

**DOI:** 10.1098/rsos.180506

**Published:** 2018-08-15

**Authors:** Jinhyeon Kang, Sanggyu Yim

**Affiliations:** Department of Chemistry, Kookmin University, Seoul 02707, South Korea

**Keywords:** nickel cobalt sulfide, alternate-dip-coating, supercapacitor, nanostructured electrode

## Abstract

Nanostructured nickel cobalt sulfide (NiCo_2_S_4_) electrodes are successfully fabricated using a simple alternate-dip-coating method. The process involves dipping a TiO_2_ nanoparticles-covered substrate in a nickel/cobalt precursor solution and sulfur precursor solution alternately at room temperature. The fabricated bimetallic sulfide electrode exhibits a synergetic improvement compensating for the disadvantages of the two single metal sulfide electrodes, i.e. the poor cycle stability of the nickel sulfide electrode and the low specific capacitance (*C*_sp_) of the cobalt sulfide electrode. The two capacitive properties are optimized by adjusting the ratio of nickel and cobalt concentrations in the metal precursor solution, reaching a *C*_sp_ of 516 F g^−1^ at a current density of 1 mA cm^−2^, with its retention being 99.9% even after 2000 galvanostatic charge–discharge cycles.

## 1. Introduction

Supercapacitors have attracted increasing attention as a next-generation energy storage device because of their excellent capacitive characteristics such as a rapid charge–discharge process, large specific capacitance (*C*_sp_) and high power density [1–3]. Supercapacitors are generally categorized into two types according to their energy storing mechanism, electrical double layer capacitors (EDLCs) and pseudocapacitors [2–4]. Recently, the pseudocapacitors using fast Faradaic charge-transfer reactions have been studied extensively due to their superior capacitive performances compared to EDLCs. The electrode materials generally used for the pseudocapacitors include transition metal oxides (TMOs) and conductive polymers [5]. Transition metal sulfides (TMSs) have also been studied recently because of their superior capacitive properties to corresponding TMOs such as high mechanical and thermal stability, high electrical conductivity and rich redox reactions [6]. Among the various TMSs, nickel sulfide (NiS_x_) has been the most widely studied due to its large theoretical capacitance, high electrical conductivity, eco-friendly properties and affordable prices [7–11]. However, easy agglomeration and pulverization while repeating the charge–discharge process hamper the practical application of NiS_x_ electrodes [7,12]. The nanostructuring of the electrodes is therefore essential; however, conventional nanostructuring techniques such as hydrothermal or solvothermal processes require harsh reaction conditions in an autoclave at high temperature for a long time [8,13–16]. To solve these problems, we recently introduced a significantly simpler nanostructuring technique, the so-called alternate-dip-coating method for fabricating nanostructured NiS electrodes [17]. The fabricated NiS nanostructured electrode exhibited a significantly improved specific capacitance and voltammetric response compared to the NiS planar film electrode. However, the *C*_sp_ retention of the NiS nanostructured electrode after 1000 charge–discharge cycles was still only approximately 60%. This low cycle stability is the intrinsic problem of NiS electrodes that needs to be overcome for their practical use [18–20]. On the other hand, a cobalt sulfide (CoS_x_) electrode was reported to have considerably larger cycle stability although its specific capacitance was quite lower than that of the NiS electrode. We therefore expected that the combination of these two TMSs would lead to a synergetic enhancement in capacitive properties, especially in cycle stability. The improved capacitive properties of the NiCo_2_S_4_ electrodes compared to the single metal sulfide, i.e. the NiS and Co_3_S_4_, electrodes have recently been reported [21]. However, the NiCo_2_S_4_ nanostructured electrodes were also fabricated under harsh fabrication conditions, mostly using a hydrothermal or solvothermal technique [21–23].

In this work, the nanostructured bimetallic nickel cobalt sulfide (NiCo_2_S_4_) electrode was more simply and successfully fabricated by an alternate-dip-coating method and its electrochemical properties were investigated. While the specific capacitance of the NiCo_2_S_4_ electrode was positioned in between the *C*_sp_ values of the NiS and Co_3_S_4_ electrodes, the cycle stability was dramatically improved, exhibiting over 99% *C*_sp_ retention even after 2000 charge–discharge cycles.

## 2. Material and methods

### 2.1. Fabrication of nanostructured metal sulfide electrodes

Nickel acetate (Ni(Ac)_2_, 99%), cobalt acetate (Co(Ac)_2_, 99%) and sodium sulfide (Na_2_S, 98%) were purchased from Aldrich. The process for the alternate-dip-coating method and fabrication of the nanostructured NiS electrodes are described elsewhere [17]. The nanostructured Co_3_S_4_ electrode was fabricated using the same process. Briefly, a fluorine-doped tin oxide (FTO)-coated glass substrate was subjected to UV-O_3_ cleaning for 5 min. A 200 nm thick porous TiO_2_ (p-TiO_2_) layer was formed on the cleaned substrate by spin-coating of commercial TiO_2_ nanoparticle (NP) paste (90-T, Dyesol) diluted in ethanol (1 : 6 weight ratio) at 2500 r.p.m., followed by annealing at 300°C for 1 h. The TiO_2_-deposited FTO substrate was placed in a 0.15 M Co(Ac)_2_ methanol/water solution and a 0.15 M Na_2_S methanol/water solution for 3 min each, washed with distilled water and blown with N_2_ gas. This alternate-dip-coating cycle was repeated seven times. For the fabrication of nanostructured NiCo_2_S_4_ electrodes, a 0.0375 M Ni(Ac)_2_ and 0.075 M Co(Ac)_2_ mixed solution was used as a metal precursor solution, and the other process was carried out in the same manner. Finally, the nanostructured metal sulfide electrodes were annealed at 200–350°C for 1 h. The deposit weights of the metal sulfides were determined by a quartz crystal microbalance (QCM, Stanford Research System QCM 2000).

### 2.2. Characterization

The crystalline structure and surface morphology of the pristine and TMS-coated p-TiO_2_ layers were characterized by X-ray diffraction (XRD, Philips PW1827) and a field emission scanning electron microscope (FE-SEM, JEOL JSM-7410F, JEOL Ltd), respectively. The electrochemical properties of the electrodes were evaluated by cyclic voltammetry (CV) and the galvanostatic charge–discharge (GCD) technique in a 2.0 M aqueous KOH solution at room temperature using a cyclic voltammeter (ZIVE SP2, WonATech). The measurements were performed in a three-electrode electrochemical cell in which the metal sulfide electrodes were used as a working electrode, a platinum plate was used as a counter electrode and Ag/AgCl (in 3.0 M KCl) was used as a reference electrode.

## 3. Results and discussion

**Figure 1.**
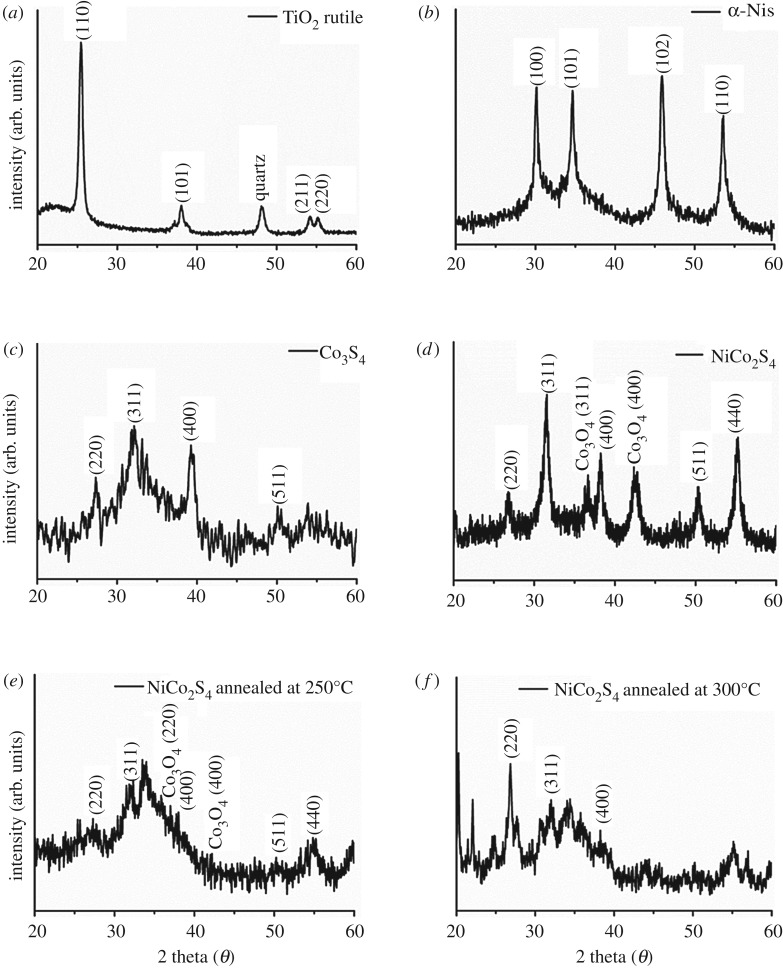
Powder XRD patterns of (*a*) pristine, (*b*) NiS-coated, (*c*) Co_3_S_4_-coated and (*d*) NiCo_2_S_4_-coated p-TiO_2_ nanoparticles layer. The annealing temperatures for the NiS, Co_3_S_4_ and NiCo_2_S_4_ layer were 300°C, 200°C and 350°C, respectively. The XRD patterns of NiCo_2_S_4_-coated p-TiO_2_ nanoparticles layers annealed at (*e*) 250°C and (*f*) 300°C are also shown.

**Figure 2.**
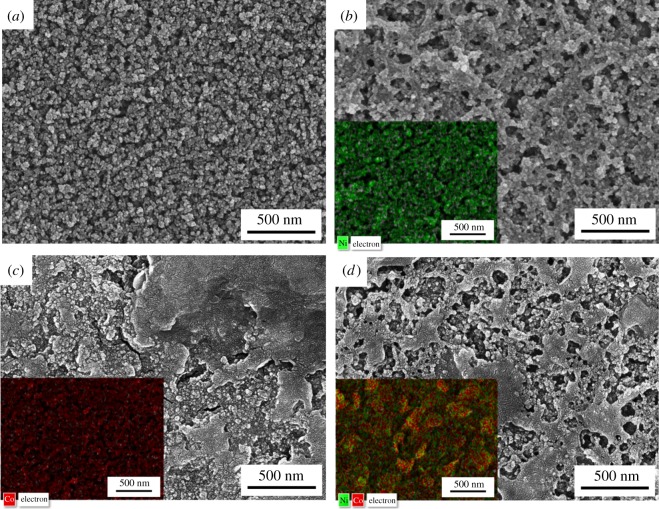
Surface FE-SEM images of (*a*) pristine, (*b*) NiS-, (*c*) Co_3_S_4_- and (*d*) NiCo_2_S_4_-coated p-TiO_2_ layer. The surface SEM-mapping images for the Ni (green) and Co (red) atoms are also shown in the insets of (*b*–*d*).

First, the p-TiO_2_ layer was prepared by the spin-coating of TiO_2_ nanoparticles with an average diameter of 20 nm, followed by sintering at 300°C for 1 h. The crystalline structure of the fabricated p-TiO_2_ layer was confirmed as a rutile structure as shown in figure 1*a*. The metal sulfide thin films were formed on the p-TiO_2_ layer by the alternate-dip-coating method in a corresponding metal precursor solution and sulfur precursor solution for 3 min each. For the deposition of the single metal sulfide thin films, i.e. NiS and Co_3_S_4_ thin films, Ni(Ac)_2_ and Co(Ac)_2_ solution were used as a metal precursor solution, respectively. The bimetallic sulfide, i.e. NiCo_2_S_4_, thin films were also prepared aiming for the synergetic improvement of the two single metal sulfide electrodes. For this preparation, a solution containing both Ni(Ac)_2_ and Co(Ac)_2_ was used as a metal precursor solution. The concentration of each precursor was adjusted stoichiometrically. After finishing the alternate-dip-coating cycles, the electrode was annealed to enhance the crystallinity of the active materials. From XRD patterns, it was observed that the nickel sulfide (figure 1*b*) and cobalt sulfide (figure 1*c*) film were composed of α-phase NiS (JCPDS card no. 02-1280) and Co_3_S_4_ (JCPDS card no. 75-1561) crystallites, respectively. The bimetallic sulfide film is composed of mainly NiCo_2_S_4_ (JCPDS card no. 20-0782) and a small amount of Co_3_O_4_ (JCPDS card no. 42-1467) as shown in figure 1*d*. The annealing temperature of the NiCo_2_S_4_ electrodes was fixed at 350°C in this work because the annealing at a lower temperature led to less crystallinity of the film as shown in figure 1*e*,*f*. Surface FE-SEM images of the electrodes are shown in figure 2. Before the deposition of the metal sulfides, the TiO_2_ nanoparticles with an average diameter of 20 nm were clearly observed in figure 2*a*. The number of alternate-dip-coating cycles for the three metal sulfide electrodes was fixed at seven. Deposit weights estimated by QCM measurements for the NiS, Co_3_S_4_ and NiCo_2_S_4_ were approximately 33, 90 and 53 µg cm^−2^, respectively. It was observed that the surface and interspace of the p-TiO_2_ nanoparticles were almost covered with metal sulfides after seven cycles of the deposition for all three electrodes. The elemental SEM-mapping results for Ni and Co atoms are also shown in the insets of figure 2. The green and red colours represent the Ni and Co atoms, respectively. As shown in the inset of figure 2*b*, the nickel atoms were well spread over the p-TiO_2_ layer for the NiS electrode. Likewise, Co atoms were also well spread over the p-TiO_2_ layer for the Co_3_S_4_ electrode (figure 2*c*). In the case of the NiCo_2_S_4_ electrode, Ni and Co atoms were observed to be uniformly spread over the p-TiO_2_ layer, as shown in the inset of figure 2*d*.

**Figure 3.**
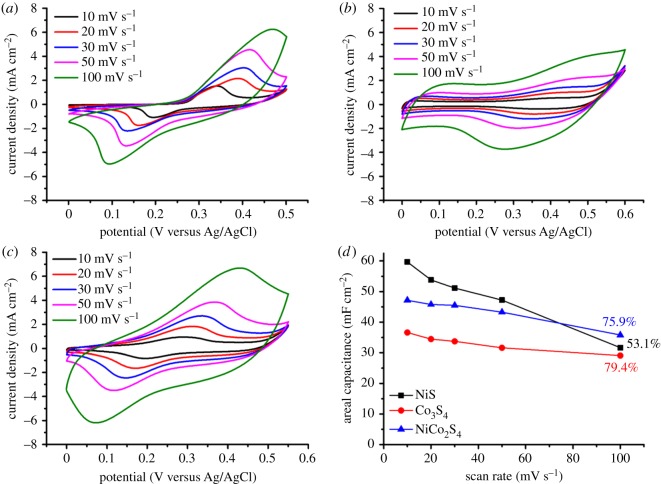
CV curves measured at various scan rates for the (*a*) NiS, (*b*) Co_3_S_4_ and (*c*) NiCo_2_S_4_ electrodes. The *C*_areal_ values calculated from the CV measurements are plotted as a function of the scan rate in (*d*).

The electrochemical performance of the three metal sulfide electrodes was estimated by CV measurements in a 2.0 M KOH aqueous solution. The potential windows were set differently for each active material, i.e. 0**–**0.5 V for the NiS, 0**–**0.6 V for the Co_3_S_4_ and 0**–**0.55 V for the NiCo_2_S_4_ electrodes. Figure 3*a–c* shows CV curves of the three electrodes at various scan rates from 10 to 100 mV s^−1^. The areal capacitance (*C*_areal_) values were calculated with the following equation:

3.1 $$C_{\mathrm{areal}}=\frac{\int J\text{d}V}{\Delta V(\text{d}V/dt)},$$

where *J* (mA cm^−2^) is the current density, Δ*V* (V) is the voltage range and d*V*/d*t* (mV s^−1^) is the scan rate. As the scan rate increased, the oxidation and reduction peak shifted to a more positive and more negative potential, respectively, which is probably caused by increased polarization at the elevated scan rates [24,25]. Figure 3*d* shows the plots of the *C*_areal_ values as a function of the scan rate for the three metal sulfide electrodes. At a scan rate of 10 mV s^−1^, the NiS-coated p-TiO_2_ electrode showed the highest *C*_areal_ value of 59.7 mF cm^−2^. The Co_3_S_4_-coated p-TiO_2_ electrode had the lowest *C*_areal_ value of 36.6 mF cm^−2^. In the case of the NiCo_2_S_4_-coated p-TiO_2_ electrode, the *C*_areal_ value was 47.2 mF cm^−2^, which is approximately an average value of the two single metal sulfide electrodes. However, at a scan rate of 100 mV s^−1^, the NiCo_2_S_4_-coated p-TiO_2_ electrode showed a higher *C*_areal_ value than those of the single metal sulfide electrodes. The *C*_areal_ retention with respect to the value at the scan rate of 10 mV s^−1^ was 75.9%. In contrast, the NiS-coated p-TiO_2_ electrode showed the lowest *C*_areal_ retention of 53.1%. The improved voltammetric response of the NiCo_2_S_4_ electrode is probably due to the higher electrical conductivity of bimetallic sulfides compared to corresponding single metal sulfides [26].

**Figure 4.**
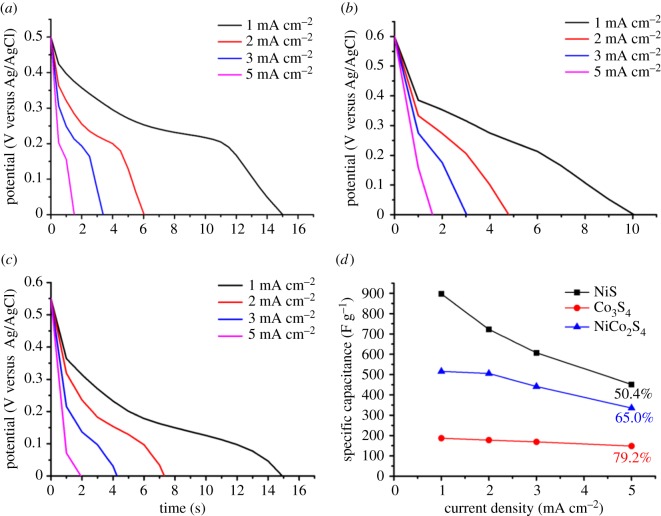
Galvanostatic discharge curves measured at various current densities for the (*a*) NiS, (*b*) Co_3_S_4_ and (*c*) NiCo_2_S_4_ electrodes. The *C*_sp_ values calculated from the discharge curves are plotted as a function of the current density in (*d*).

The same tendency was also observed in the GCD measurement. The discharge curves for the three metal sulfide electrodes measured at various current densities are shown in figure 4*a*–*c*. The *C*_sp_ values are calculated with the following equation:

3.2 $$C_{\mathrm{sp}}=\frac{I\Delta t}{m\Delta V},$$

where *I* (A) is the discharge current, *m* (g) is the deposited weight of the metal sulfides, Δ*t* (s) is the total discharge time and Δ*V* (V) is the voltage drop during the discharge [27]. The calculated *C*_sp_ values of the NiS, Co_3_S_4_ and NiCo_2_S_4_ electrodes at a current density of 1 mA cm^−2^ are 896.9, 187.0 and 515.7 F g^−1^, respectively. As expected, the NiS electrode showed the highest specific capacitance, and the *C*_sp_ value of the NiCo_2_S_4_ electrode was placed between the values of the NiS and Co_3_S_4_ electrodes. The plots of the calculated *C*_sp_ values of the three metal sulfide electrodes at various current densities are shown in figure 4*d*. Similar to the rate capabilities obtained from the CV curves, the NiS-coated p-TiO_2_ electrode had the lowest *C*_sp_ retention of 50.4% at a current density of 5 mA cm^−2^. At the same measurement condition, the *C*_sp_ retention of the Co_3_S_4_ electrode was 79.2%. The *C*_sp_ retention of the NiCo_2_S_4_ electrode, 65.0%, was positioned between the values of the two single metal sulfide electrodes. The NiCo_2_S_4_ electrode exhibited an energy density of 21.7 W h kg^−1^ at a power density of 5200 W kg^−1^, as calculated using the following formulae:

3.3 $$E=\frac{5C_{\mathrm{sp}}{\left(\Delta V\right)}^{2}}{36}$$

and

3.4 $$P=\frac{3600E}{\Delta t},$$

where *C*_sp_ (F g^−1^) is the specific capacitance obtained from the GCD measurements at a current density of 1 mA cm^−2^, Δ*V* (V) is the applied potential window and Δ*t* (s) is the discharge time [28,29].

**Figure 5.**
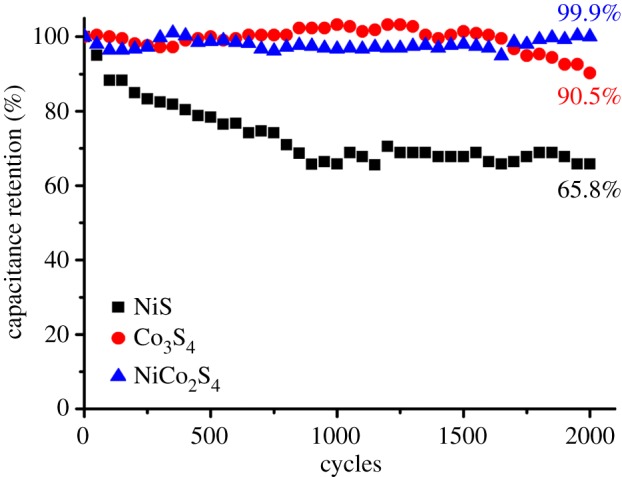
Plots of the *C*_sp_ retention for the three metal sulfide electrodes as a function of the number of galvanostatic charge–discharge cycles. The current density was fixed to 3.0 mA cm^−2^ during the measurements.

The cycle stability of the metal sulfide electrodes was estimated by repeating continuous galvanostatic charge–discharge cycles at a constant current density of 3 mA cm^−2^. Figure 5 represents the *C*_sp_ retentions of the three electrodes as a function of the number of GCD cycles. The NiS electrode had the lowest retention of approximately 66% after 2000 charge–discharge cycles. In contrast, the Co_3_S_4_ and NiCo_2_S_4_ electrodes had *C*_sp_ values that barely changed during the 2000 cycles. This indicates that after 2000 charge–discharge cycles, the *C*_sp_ value of the NiCo_2_S_4_ electrode exceeds the *C*_sp_ value of the NiS electrode, although at the initial stage, the *C*_sp_ value of the NiS electrode is approximately 1.5 times larger than that of the NiCo_2_S_4_ electrode. The superior electrochemical properties such as the electrical conductivity, specific capacitance and cycle stability of bimetallic sulfides to those of the corresponding single metal sulfides have also been reported previously [26,30,31]. Overall, the simple fabrication and synergetic improvements of the nanostructured NiCo_2_S_4_ electrodes demonstrated in this study can be applied to manufacturing efficient metal sulfide electrodes for supercapacitors.

## 4. Conclusion

The nanostructured NiS, Co_3_S_4_ and NiCo_2_S_4_ electrodes for supercapacitors were simply fabricated by alternately dipping a TiO_2_ nanoparticles-covered FTO substrate into a metal and a sulfur precursor solution. When the Ni(Ac)_2_ and Co(Ac)_2_ solutions and their 1 : 2 (mole/mole) mixed solution were used as a metal precursor solution in the process, the α-NiS, Co_3_S_4_ and NiCo_2_S_4_ electrodes were fabricated, respectively. A maximum *C*_sp_ value of 897 F g^−1^ at a current density of 1 mA cm^−2^ was obtained for the nanostructured NiS electrode, although it had the lowest capacitance retention of 66% after 2000 GCD cycles. In contrast, the Co_3_S_4_ electrode had a significantly higher voltammetric response and cycle stability although its *C*_sp_ value was quite low at 187 F g^−1^. The bimetallic sulfide electrode, i.e. NiCo_2_S_4_ electrode, was then fabricated for synergetic improvement from the properties of the two single metal sulfide electrodes. As expected, the NiCo_2_S_4_ electrode showed a dramatic increase in the voltammetric response and cycle stability with a slightly reduced *C*_sp_ value of 516 F g^−1^, compared to the NiS electrode. The *C*_sp_ retention of the nanostructured NiCo_2_S_4_ electrode was approximately 100% with respect to its initial value even after 2000 GCD cycles.

## Data accessibility

Our data are deposited at Dryad (http://dx.doi.org/10.5061/dryad.qs3jn16) [32].
